# Deep sequencing reveals new roles for MuB in transposition immunity and target-capture, and redefines the insular Ter region of *E. coli*

**DOI:** 10.1186/s13100-020-00217-9

**Published:** 2020-07-09

**Authors:** David M. Walker, Rasika M. Harshey

**Affiliations:** Department of Molecular Biosciences, University of Texas at Austin, Austin, TX 78712 USA

## Abstract

**Background:**

The target capture protein MuB is responsible for the high efficiency of phage Mu transposition within the *E. coli* genome. However, some targets are off-limits, such as regions immediately outside the Mu ends (*cis*-immunity) as well as the entire ~ 37 kb genome of Mu (Mu genome immunity). Paradoxically, MuB is responsible for *cis*-immunity and is also implicated in Mu genome immunity, but via different mechanisms. This study was undertaken to dissect the role of MuB in target choice in vivo.

**Results:**

We tracked Mu transposition from six different starting locations on the *E. coli* genome, in the presence and absence of MuB. The data reveal that Mu’s ability to sample the entire genome during a single hop in a clonal population is independent of MuB, and that MuB is responsible for *cis*-immunity, plays a minor role in Mu genome immunity, and facilitates insertions into transcriptionally active regions. Unexpectedly, transposition patterns in the absence of MuB have helped extend the boundaries of the insular Ter segment of the *E. coli* genome.

**Conclusions:**

The results in this study demonstrate unambiguously the operation of two distinct mechanisms of Mu target immunity, only one of which is wholly dependent on MuB. The study also reveals several interesting and hitherto unknown aspects of Mu target choice in vivo, particularly the role of MuB in facilitating the capture of promoter and translation start site targets, likely by displacing macromolecular complexes engaged in gene expression. So also, MuB facilitates transposition into the restricted Ter region of the genome.

## Introduction

**Fig. 1:**
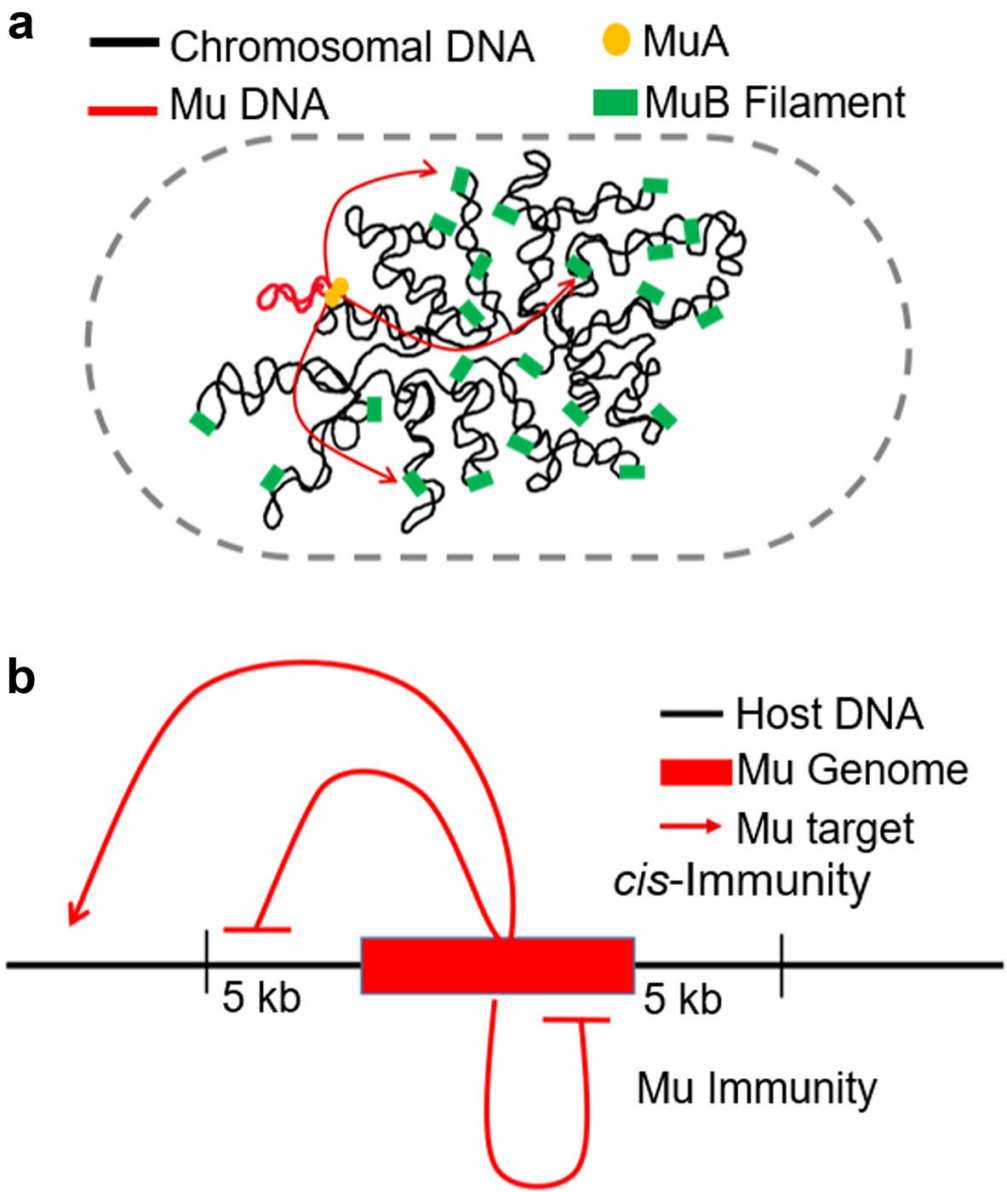
Mu transposition and target immunity. **a**. The transposase MuA pairs Mu ends and introduces single-strand nicks, joining these to MuB-captured target DNA (red arrows). MuB binds DNA non-specifically, polymerizing in short filaments, and increases the catalytic efficiency of target capture. **b**. *Cis-*immunity and Mu genome immunity. *Cis-*immunity is characterized by the lack of insertions outside Mu ends (typically within 5 kb), and Mu genome immunity by absence of insertion anywhere within the ~ 37 kb Mu genome

Phage Mu uses transposition to amplify its genome ~ 100-fold during its lytic cycle in *E. coli*, making it the most efficient transposable element (TE) described to date [1–3] (Fig. 1a). Mu transposes by a nick-join pathway, where assembly on Mu ends of a six-subunit MuA transposase complex (transpososome) is followed by introduction of nicks at both ends; the liberated 3′-OH groups at each end then directly attack phosphodiester bonds spaced 5 bp apart in the target DNA, covalently joining Mu ends to the target [4]. The resulting branched Mu-target joint is resolved by replication, duplicating the Mu genome after every transposition [5]. At the end of the lytic cycle, Mu copies are excised for packaging by a headful mechanism that cuts and packages host DNA on either side of Mu [1, 6]. The latter finding has been exploited to examine target site preference in vivo by sequencing the flanking host DNA packaged in Mu virions [7, 8].

**Table 1:** Key Resources Table

REAGENT or RESOURCE	SOURCE	IDENTIFIER
**Chemicals, Peptides, and Recombinant Proteins**		
HinP1	New England Biolabs	R0124S
Phusion Polymerase	New England Biolabs	M0530S
Critical Commercial Assays		
Wizard Genomic DNA Purification Kit	Promega	A1120
Quick Ligation Kit	New England Biolabs	M2200L
Qiaquick PCR Cleanup Kit	Qiagen	28,106
Axygen™ AxyPrep Mag™ PCR Clean-up Kits	Thermo Scientific	14–223-227
**Deposited Data**		
Genomic Sequencing Data	This Study	https://www.ncbi.nlm.nih.gov/sra/PRJNA597349
**Organisms/Strains**		
MP1999 Mu*cts B::kan.* Mu insertion between nts 3,652,046–3,652,051	(Saha et al. 2013) [28]	ZL530
MG1655 with Mu*cts*:*cat* at nt 4,203,381 (Ori-Mu)	(Walker et al. 2020) [29]	DMW11
MG1655 with Mu*cts:cat* at nt 263,131 (OPR-Mu)	(Walker et al. 2020) [29]	DMW15
MG1655 with Mu*cts:cat* at nt 3,339,450 (OPL-Mu)	(Walker et al. 2020) [29]	DMW22
MG1655 with Mu*cts*:*cat* at nt 2,555,144 (TPL-Mu)	(Walker et al. 2020) [29]	DMW24
MG1655 with Mu*cts*:*cat* at nt 792,226 (TPR-Mu)	(Walker et al. 2020) [29]	DMW33
MG1655 with Mu*cts*:*cat* at nt 1,657,887 (Ter-Mu)	(Walker et al. 2020) [29]	DMW57
DMW57, Mu*cts*:*cat B::kan*	This study, DMW57, ZL530	DMW300
DMW33, Mu*cts*:*cat B::kan*	This study, DMW33, ZL530	DMW301
DMW24, Mu*cts*:*cat B::kan*	This study, DMW24, ZL530	DMW302
DMW22*,* Mu*cts*:*cat B::kan*	This study, DMW22, ZL530	DMW303
DMW15, Mu*cts*:*cat B::kan*	This study, DMW15, ZL530	DMW304
DMW11, Mu*cts*:*cat B::kan*	This study, DMW11 ZL530	DMW305
**Software and Algorithms**		
MAPS (Python)	This Study	https://github.com/dmwalker/MuSeq
BWA-MEM	Li H., 2013	https://sourceforge.net/projects/bio-bwa/

The B protein of Mu (MuB), a non-specific DNA-binding protein and AAA+ ATPase, is essential for the efficient capture and delivery of the target to the transpososome via MuB-MuA interaction; MuB also plays critical roles at all stages of transposition by allosterically activating MuA (see [3, 9]). MuB forms ATP-dependent helical filaments, with or without DNA [10–12]. Because of a mismatch between the helical parameters of the MuB filament and that of the bound DNA, it has been proposed that the DNA at the boundary of the MuB filament deforms, creating a DNA bend favored by MuA as a target [11, 13, 14]. While most TEs display some degree of target selectivity [15], Mu is perhaps one of the most indiscriminate, with a fairly degenerate 5 bp target recognition consensus [7, 8, 16, 17]. Even though MuB facilitates target selection, recognition of the 5-bp target site is a property of MuA, and is independent of MuB.

Several bacterial TEs, including members of the Tn*3* family, Tn*7*, and bacteriophage Mu, display transposition immunity [15, 18–22]. These elements avoid insertion into plasmid DNA molecules that already contain a copy of the transposon (a phenomenon called *cis*-immunity), and it is thought that this form of self-recognition must also provide protection against self-integration (TE genome immunity) (Fig. 1b). While *cis*-immunity in vitro extends over the entire plasmid harboring the TE, it does not provide protection to the entire bacterial genome on which the TE is resident, but can extend over large distances from the chromosomal site where it is located. In vitro studies with mini-Mu donor plasmids provided the first molecular insights into the *cis*-immunity phenomenon [9, 23]. Ensemble and single-molecule experiments showed that MuB bound to DNA dissociates upon interaction in *cis* with MuA bound to the Mu ends, resulting in depletion of MuB near the vicinity of Mu ends, making the depleted region a poor target for new insertions [24, 25]. It was assumed that this mechanism also protects DNA inside Mu ends. *Cis*-immunity has been observed in vivo, appearing strongest around 5 kb outside the Mu ends, and decaying gradually between 5 and 25 kb [26, 27].

The proposition that *cis*-immunity also prevents self-integration is a reasonable one for TEs whose size is smaller than the range over which this immunity extends. For Mu, *cis*-immunity has been tested over a 2–3 kb range in vitro using mini-Mu plasmids, and found to be strongest around 5 kb in vivo as stated above. The range of immunity seen in vivo would not be expected to effectively protect the 37 kb Mu genome by the *cis*-immunity mechanism, as was indeed demonstrated to be the case [27]. Therefore, a distinct ‘Mu genome immunity’ mechanism was proposed to explain the lack of self-integration. Unlike the *cis*-immunity mechanism, which requires removal of MuB from DNA adjacent to Mu ends, MuB was observed to bind strongly within the Mu genome during the lytic cycle, suggesting that the mechanism of Mu genome-immunity must be different from that of *cis*-immunity [27]. ChIP experiments revealed sharply different patterns of MuB binding inside and outside Mu, leading to a proposal that the Mu genome is segregated into an independent chromosomal domain in vivo [27]; this proposal was confirmed by Cre-*loxP* recombination and 3C experiments for Mu prophages at two different *E. coli* chromosomal locations [28]. A model for how the formation of an independent “Mu domain” might nucleate polymerization of MuB on the genome, forming a barrier against self-integration, was proposed [27].

The present study investigates the role of MuB in the three diverse functions discussed above - target capture, *cis-*immunity, and Mu genome immunity in vivo. Through comparison of insertion patterns of wild-type (WT) and ΔMuB prophages placed at six different locations around the *E. coli* genome, we show that *cis*-immunity depends on MuB, while Mu genome immunity is only mildly breached in its absence. The data also reveal a previously unappreciated role for MuB in facilitating Mu insertion into transcriptionally and translationally active regions. An unanticipated outcome of this study is the finding that the Ter segment of the *E. coli* genome, which is more isolated from the rest of the genome, is larger than previously estimated.

## Results and discussion

### Mu samples the entire *E. coli* genome even in the absence of MuB, helping define new boundaries for the Ter region

We recently exploited the DNA-DNA contact mechanism of phage Mu transposition to directly measure in vivo interactions between genomic loci in *E. coli* [29]. Thirty-five independent Mu prophages located throughout the genome were induced to go through one round of transposition. The data showed that in a clonal population, Mu is able to access the entirety of the genome with roughly equal probability regardless of its starting genome location, suggesting widespread contacts between all regions of the chromosome. The data led us to conclude that the chromosome is well-mixed and shows a ‘small world’ behavior, where any particular locus is roughly equally likely to be in contact with any other locus. The exception was the Ter region, reported by Mu as being less well-mixed than the rest of the genome.

**Fig. 2:**
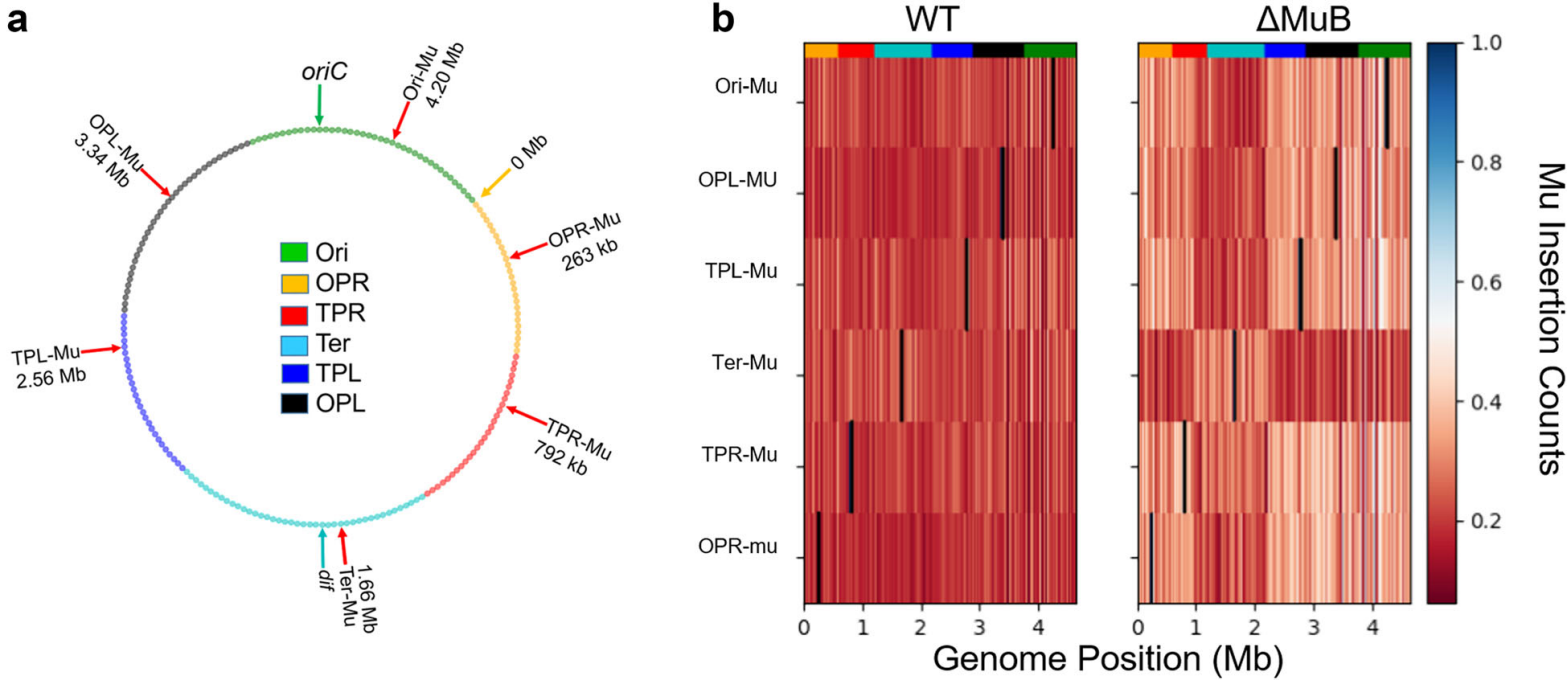
Mu samples the entire genome regardless of the presence of MuB. **a**. The six starting prophage locations on the *E. coli* genome monitored in this study are indicated by red arrows (see Table 1 for their exact locations). These locations were chosen because they are distributed throughout the chromosome, and therefore ideally suited for sampling features across the genome. *oriC* in the Ori region is the site where bi-directional replication begins (green arrow), terminating at the *dif* site, exactly opposite to *oriC* within the Ter region (cyan arrow). OPL, Ori proximal left; OPR, Ori proximal right; TPL, Ter proximal left; TPR, Ter proximal right. The boundaries of the various colored regions are taken from [31]. **b**. The genome was partitioned into 200 equally sized bins (**a**), and the normalized number of unique insertions into each bin for each prophage was computed, as displayed by the color bar on the right. The highest number of unique insertions for any non-starting bin was ~ 8000 insertions corresponding to just under 1.0. Each starting bin position can be identified by the dark blue bars. The multi-color strip on top of each panel corresponds to chromosomal regions shown in **a**. The Ter region (cyan) as explored by the ΔMuB prophages is 217 kbp larger than earlier estimates [31]. This is recognizable as a square block of lighter red insertions in the Ter-ΔMuB prophage, which lines up with identical blocks of darker red insertions in the other five ∆MuB prophages

While MuB is essential for target capture in vitro [4], transposition is still detectable in vivo in the absence of MuB at an efficiency nearly two orders of magnitude lower than WT [30]. To examine how MuB influences the target selection in vivo, we monitored insertion patterns of a subset (six) of the Mu prophages used in the original study [29] (Fig. 2a), after a single round of transposition, in the presence and absence of MuB (WT vs ΔMuB) (Fig. 2b). For analysis, the genome was partitioned into 200 equally sized bins (each bin ~ 23.2 kb) (Fig. 2a). To generate sufficient insertion resolution, transpositions were analyzed using a target enrichment protocol [29] and deep sequencing of 10 million reads or more. Due to lower transposition frequencies of ΔMuB prophages, these were sampled ~ 50% more with a 15 million read depth. The data plotted in Fig. 2b show similar insertion profiles for both WT and ΔMuB throughout the genome after normalizing to the read depth for both prophages. Thus, like WT, the ΔMuB prophages transpose to every bin of the genome in a clonal population, allowing us to conclude that the ability of Mu to sample the entirety of the genome in one transposition event is independent of MuB.

The color-coded map of the *E. coli* genome shown in Fig. 2a depicts the length and boundaries of chromosomal regions deduced by prior methodologies to be either unreactive or partially reactive with the other regions [31]. With the exception of Ter, the Mu methodology failed to detect all such boundaries [29]. The Ter region has unique properties shaped by the activity of MatP [32] and the condensin MukBEF [33, 34], and has been shown by several methodologies to be more isolated from the rest of the chromosome [29, 35, 36]. Comparison of WT vs ΔMuB insertion patterns supported this conclusion while revealing more details. For example, the ΔMuB prophage located in Ter (Ter-Mu) had > 40% of its total insertions occur within the Ter region, which only comprises ~ 20% of the genome (light red profile). While Ter-ΔMuB prophage sampled the DNA around its starting location more efficiently than it did the rest of the genome, the ΔMuB prophages at the five other locations showed a converse pattern in that they could not access Ter as easily (dark red profile). The latter prophages had < 15% of their total insertions within Ter. Comparison of both the outgoing and incoming ΔMuB profiles all lined up precisely, giving us a clearer view of the boundaries flanking Ter. According to Valens et al. [31], the Ter region extends from nucleotide position 1128 kb (26′ on the genetic map) to 2038 kb (47′). According to the transposition patterns of ΔMuB prophages, the Ter region extends from nucleotide position 911 kb (21′) to 2200 kb (47′), expanding the left boundary by more than 217 kb (Fig. 2b). We note that ΔMuB prophages did not reveal other boundaries (as demarcated by the colored segments in Fig. 2a) proposed by prior methodologies [31].

Why does such a defined Ter segment emerge only in MuB-deficient prophages? Given that the ΔMuB prophage in Ter had no trouble sampling within Ter, but that the other ΔMuB prophages did have difficulty inserting here, we suggest that the answer lies in the existence of some special feature at the Ter boundaries that isolates Ter. MatP, which binds to specific matS sequences distributed within Ter [32], has been shown to functionally exclude the SMC/condensin complex MukBEF from Ter [33]. Fluorescence experiments have shown that the extent to which MatP organizes Ter and excludes MukBEF ranges from 852 kb to 2268 kb [34], which is much more in line with our estimates of Ter in the ΔMuB prophages. To our knowledge, MatP is not itself enriched at the Ter boundaries [34]. Perhaps, as an SMC complex, with assistance from other proteins, MukBEF tethers the two chromosomal arms at the Ter boundary, preventing Ter from mixing with the rest of the genome. Given that WT prophages are not as impaired as ΔMuB prophages in sampling Ter, it follows that MuB must weaken the Ter boundary conditions. The property of MuB to nucleate as helical filaments on DNA [11], may be responsible for displacing the boundary-guards. These results imply that the Ter segment is even less well-mixed than determined in the study utilizing WT Mu [29].

### Highly transcribed regions are only accessible to transposition in the presence of MuB

**Fig. 3:**
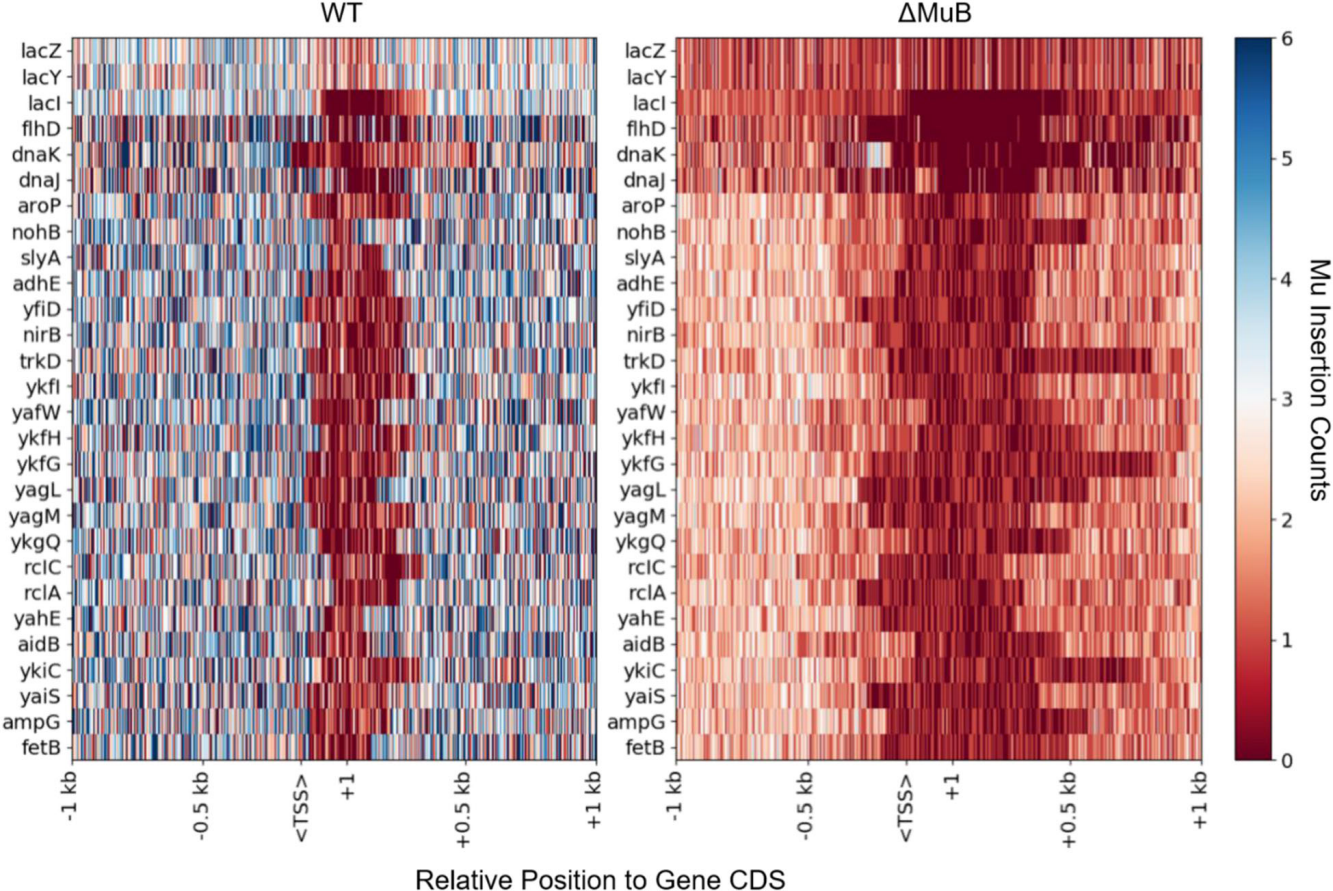
MuB is responsible for capturing target sites near highly transcribed/translated genes. Twenty-two highly transcribed genes, plus the *lac* operon, *flhD* and *dnaK-dnaJ*, were selected for comparison between WT and ΔMuB insertion patterns. For WT, transpositions were pooled from all six prophage locations with an average of 5 million reads per prophage. ΔMuB experiments pooled all six prophages with an average of 20 million reads per prophage. Each gene is oriented to where the + 1 nt of the coding sequence (CDS) of the gene starts at the tick mark labeled + 1, and downstream sequence follow to the right. Upstream regions are marked by negatively labeled tick marks. The expected transcription start site labeled <TSS> is 125 nt away from the + 1 nt site. ΔMuB prophages showed an increase in an exclusion zone starting near the + 1 nt site in nearly every single case

Two prior microarray data sets have shown a negative correlation between transcription and Mu transposition [37, 38], although one of these studies found several exceptions to this rule, and suggested that some other cellular feature controls these insertion events [38]. We examined this issue for WT and ΔMuB prophages using our higher-resolution data set. Figure 3 compiles a list of 28 genes, most of which are highly transcribed, except for the *lac* operon, which is expected to be only partially transcribed under our growth conditions. The figure also includes the flagellar master regulator gene *flhD* which has multiple promoters [39], and *dnaJ* which has no promoter and is exclusively co-transcribed with *dnaK* [40]. For all genes, the earliest identified nucleotide in the coding sequence (CDS) from the annotated genome from genebank (genid: 545778205) is defined as the + 1 nucleotide (nt) of the CDS. The data presented assume that global transcriptional levels are not affected drastically within the short time span of a single transposition event.

WT Mu had significant difficulty inserting near the + 1 nt of all active genes, in a region that extends up to 50–200 bp upstream, typically including promoter regions (TSS) [41], and 50–300 bp downstream. However, the transposition difficulty was exacerbated in ΔMuB prophages, which showed an increase in an exclusion zone starting near the TSS for transcriptionally active genes and to a lesser extent for the comparatively less transcriptionally active *lacZY*. Interestingly, two different WT Mu insertion patterns were observed within the *lac* operon, whose *lacZ* and *lacY* genes are repressed by the activity of the *lacI* repressor, which is expected to be transcribed [42]*.* The number of Mu insertions in *lacI* were roughly half those in *lacZY*, with a strong suppression of insertions around the TSS and + 1 nt region of *lacI* for WT. This observation is in agreement with the previous findings of a negative correlation between transcription and transposition.

Of six potential promoters in the *flhDC* operon that control flagellar gene transcription in *Salmonella*, only two (P1 and P5) were seen to be functional [39]. These two sites are each 200–300 bps upstream of the + 1 nt. On the other hand, the specific transcriptional start site for *dnaJ* is 2 kb away, as *dnaJ* is always co-transcribed with *dnaK*, with a small 370 nt RNA candidate *tpke11* between the two genes [40, 43]. WT prophages show a near uniform sampling across *flhD,* with reduced insertion around the TSS, while ΔMuB prophages show in addition a secondary exclusion zone upstream from the + 1 nt that encompasses both P1 and P5 promoter regions. Even though TSS is absent in *dnaJ*, WT Mu shows an insertion exclusion zone around + 1 nt of this gene. ΔMuB prophages show an exclusion zone upstream of *dnaJ* not seen in WT, around the position of *tpke11*, while revealing an unusually permissive region upstream of *dnaK*. The latter permissive region in both WT and ΔMuB corresponds to the 377 bp intergenic region between *yaaI* and the *dnaKJ* operon promoter. While this set insertion patterns overall is consistent with the negative correlation between transcription and transposition, particularly around the TSS and + 1 nt for WT, the insertion patterns in *dnaJ* reveal that the + 1 nt region presents a transposition barrier independent of the promoter region, and is likely reflective of the translation activity of the mRNA near this genomic site given that transcription and translation are coupled in bacteria. As nascent mRNA is being translated, the ribosomes could slow down the RNA polymerase enough to provide steric protection to the genomic DNA from Mu transposition.

To examine Mu insertion patterns in genes that are transcribed but not translated, we looked at both ribosomal RNA operons and tRNA genes. *E. coli* has 7 ribosomal RNA operons that are highly transcribed [44]. We observed a large variation of insertion profiles in these regions (Fig. S1). For example, the insertion frequency of WT Mu is highest in *rrnA*, uniform across the entire operon, and independent of MuB. *rrnE* and *rrnH* receive more insertions in the 23S compared to the 16S region, and are responsive to MuB. *rrnG* shows a large increase in sampling only at the 5′ end of the 16S region (note that *rrnG* is on the negative strand). There seems to be an equal level of Mu insertion between *rrnB*, *rrnC*, and *rrnD*. In the 5.3 kb window encompassing the entirety of each of these operons, over 85% of the window is comprised of coding sequences. WT transpositions into the coding sequences make up between 20 and 50% of all reported transpositions, rather than the 85% if it was entirely random. MuB mutants typically faired much worse, ranging from 5 to 10% of observed transpositions in the same area. Thus in the majority of cases, there is a significant reduction of Mu transposition into the ‘coding’ regions of the rRNA operons in the absence of MuB.

If transcriptional status determines Mu insertion efficiency as concluded from the data in Fig. 3, then the insertion patterns observed in the *rrn* operons should reflect this as well. Accordingly, *rrnA* is the least transcriptionally active. While early experiments showed little difference in expression levels between the operons in minimal media (*rrnA* actually was reported to have marginally higher expression levels [44]), more recent experiments reporting promoter activity for the *rrn* operons as measured by binding of Fis, a regulator of *rrn* transcription [45], have determined that *rrnE* has the highest level of activity in minimal media with *rrnA* having relatively low levels of promoter activity [46]. Our results are more in line with the newer data, in that Mu activity is highest within *rrnA,* and lowest near the promoter region of *rrnE* (Fig. S1). Regardless of the *rrn* operon, there seems to be a small window between the 16S and 23S subunits in each operon that is marked by an increase in insertion frequency. This window contains non-coding sequence as well various tRNA sequences. The latter are highly undersampled by Mu insertions, even when they occur elsewhere in the chromosome as discussed below.

Mu insertion patterns into 86 tRNA genes scattered throughout the *E. coli* genome [47], are shown in Figure S2. Mu shows an interesting selectivity for inserting into 30 of these genes, avoiding the region that would ultimately be the mature tRNA sequence (+ 1 to e), as exemplified by the large hole or gap with no insertions in this region through most of the WT Mu panel. Note that Mu is more actively inserting into the genomic regions associated with the 5′ leader and 3′ tailing sequences of pre-tRNA. This would suggest that there is some genomic feature (fold, DNA-binding protein) that is ultimately protecting the mature tRNA region of DNA from Mu insertion. ΔMuB prophages incidentally were less likely to insert into the entire pre-tRNA sequence, suggesting that the transcriptome machinery provided a much higher barrier of access to the ΔMuB prophages over the WT prophages. Using genome-wide transcription propensity data [48], we were able to compare the levels of transcription for each of the tRNA sequences along with the likelihood that Mu (both WT and ΔMuB) would transpose within them. Although the transcriptional information was quantitatively sparse amongst most of the tRNA genes, the accessibility of insertion into 36 tRNAs that are the lowest transcriptionally active genes, and exclusion of insertion into the highest transcriptionally active regions found within both *ileY* and *selC* (marked with red asterisks)*,* is unmistakable (Fig. S2 bottom). In these two genes, there are no insertions in regions of high transcription for either WT or ΔMuB prophages, the only insertions occurring in the lesser transcribed leader of *selC* for WT. This pattern is a general trend, there being no Mu insertions within or near any region that has a considerable amount of transcriptional activity as reported by Scholz, et al. [48].

We conclude that the level of availability of a target for Mu insertion is highly correlated with its transcriptional activity, enhanced in the presence of MuB and suppressed in its absence. The particular difficulty of WT Mu in inserting around the TSS could be a combination of an ‘open complex’ DNA at this site, occupancy by RNA polymerase, or because promoter regions are A/T rich; MuB is reported to exhibit a tendency to form larger filaments on A/T-rich DNA [10, 49]. MuB binding around promoter regions may block insertion of WT Mu there, as Mu transposition has been observed at the junction of A/T and non-A/T DNA in vitro [50], and near the vicinity rather than within, MuB-bound regions in vivo [38]. For translated genes, the evidence points to a relationship between transcriptional as well as subsequent translational activity of the mRNA in blocking Mu transposition, as demonstrated by insertion patterns around the + 1 nt position of *dnaJ*. In the case of the transcriptionally and therefore translationally inactive *lacZY* genes, we see that there is no barrier to insertion at the + 1 nt site, reinforcing this conclusion. As speculated above for the role of MuB in weakening the Ter boundary, we suggest that the filament-forming property of MuB may dislodge transcribing RNA polymerase and ribosomes from transcriptionally active DNA, collaterally increasing the availability of these macromolecular complexes for Mu morphogenesis. The most under-sampled regions on the genome are coding regions of tRNA, even though Mu is able to sample the leader sequences of the pre-tRNA coding regions, suggesting that some feature of these regions other than transcription protects them from Mu insertion.

### Target consensus in vivo

The 5-bp target recognition site for Mu transposition was derived from in vitro experiments to be 5′-CYSRG, and observed to be independent of MuB [16, 17]. In the Mu transpososome crystal structure, a hairpin bend in the target was observed, with the transpososome contacting a 20–25 target segment [13]. Preference for a bent target conformation is supported by other in vitro experiments [14, 51]. Analysis of target sequences in vitro detected symmetrical base patterns spanning a ∼ 23 to 24-bp region around the target recognition site, indicative not of an extended sequence preference, but possibly of a structural preference that might facilitate target deformation [17].

In vivo, a preference for 5′-CGG as the central triplet was derived from cloning 100 Mu-host junctions from packaged phage particles [8]. To re-examine target preference using our current data set, we pooled the insertion data totaling over 120 million targeted Mu reads for both the WT and ΔMuB constructs. We observed that in the genome, sequences with the triple-‘G’ consensus and their reverse complement were 3–4 times more abundant than the 5′-CYSRG-3′ sequences, explaining the preference for 5′-CGG in the earlier study (Fig. S3A). For WT, sequencing data suggest that there is a 7-fold preference for the 8 possible 5′-CYSRG-3′ consensus sequences over the other 1016 remaining 5-bp sequences (Fig. S3B left). This preference increases to 20-fold in ΔMuB prophages (Fig. S3B right), confirming that the consensus sequence for integration is a feature of the transposase MuA rather than a binding preference for MuB. Given that the target is severely bent in the transpososome [13], we expanded the consensus sequence search in multiple ways to determine if there were any factors such as flexible dinucleotide steps flanking the original 5-bp that impacted target selection beyond the 5-bp search. The new consensus search looked at 5′-CYSRGNN, 5′-NNCYSRG, and 5′-NCYSRGN. These expanded searches did not increase the likelihood of insertions, which generally remained within 5% of each other when normalized to genomic abundance. These observations lead us to conclude that the originally proposed 5-bp consensus as recognized by the transpososome is the largest factor in determining site insertion.

### MuB is responsible for *cis*-immunity

The *cis-*immunity phenomenon has been studied in vitro exclusively by the Mizuuchi group, from ensemble experiments with mini-Mu plasmids to single molecule experiments with tethered is λ DNA [9, 23]. A diffusion ratchet model, in which MuA-MuB interactions form progressively larger DNA loops, was proposed to explain the clearing of MuB near the vicinity of Mu ends, with eventual insertion of Mu at sites distant from the ends [24, 25].

We graphed Mu insertions flanking the ends of each starting position, by pooling information from all six prophages during the first round of transposition, as was done for all prior experiments, but we refer to here as early stage transposition (EST), to distinguish them from late stage transposition (LST) where data were collected after multiple rounds of transposition. For the LST condition, we let the experiment run for 2 h, which allowed WT to complete its lytic cycle (in ~ 50 min) and ΔMuB prophages to accumulate 5 to 10 copies of Mu on average per cell as predicted by genome abundance, assuming an even distribution of Mu copy number among the population. All six prophage strains were used for EST experiments, and one WT plus all six ΔMuB prophages for LST experiments.

**Fig. 4:**
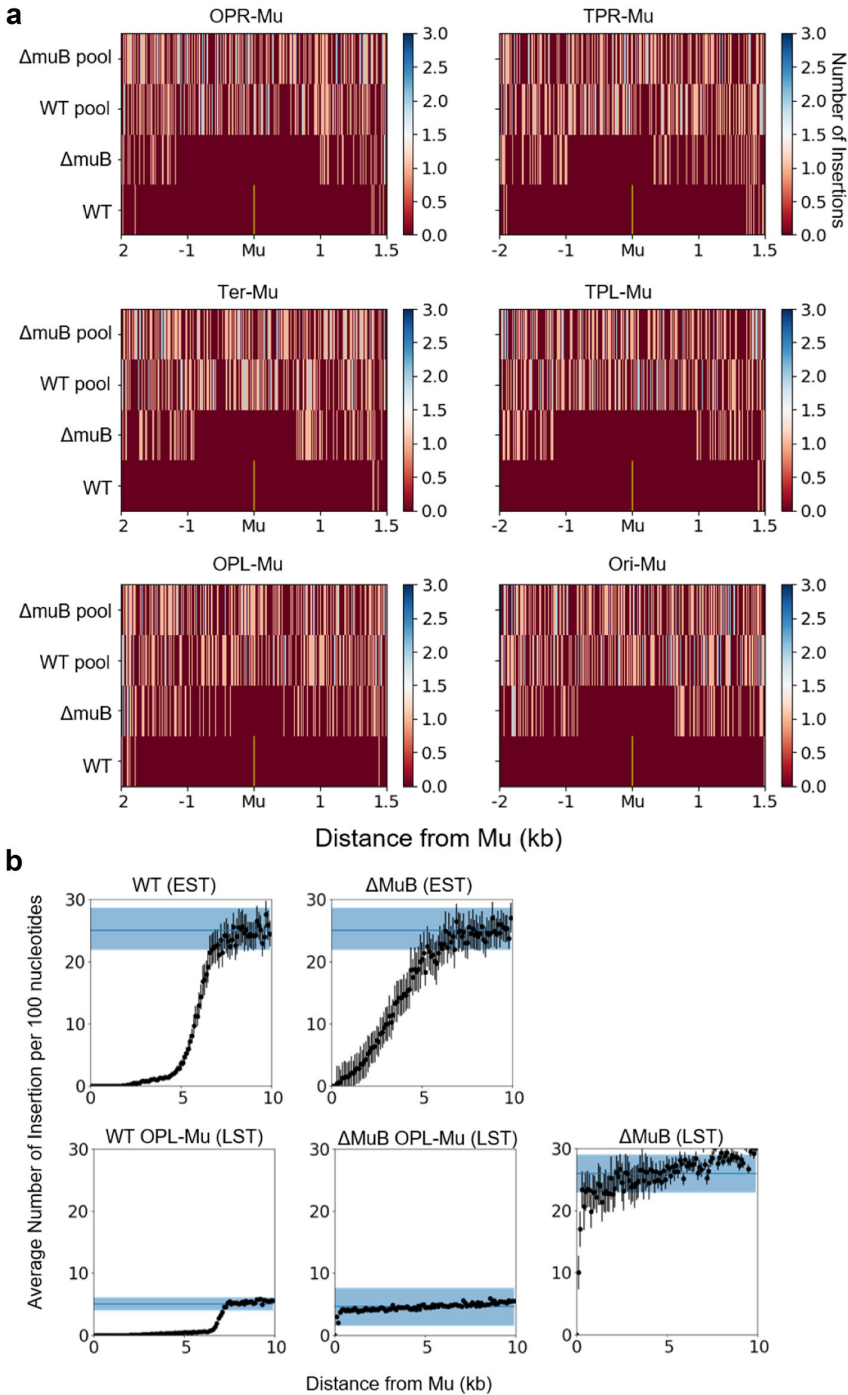
MuB is responsible for *cis*-immunity. The number of insertions near the initial starting location for each Mu prophage was tracked outside both the left and right ends of Mu during EST (early stage transposition; 15 min post-induction of transposition) and LST (late stage transposition; 2 h post-induction). **a**. The frequency of Mu insertions during EST for all six prophages, both WT and ΔMuB, under four different experimental steps (see text). Pooled experiments are frequency of insertions into that particular Mu location from the other 5 prophages, and indicate that all these particular chromosomal locations are readily transposed into in the absence of Mu. The initial position of Mu is indicated by a yellow line in the center of each plot. **b** The frequency of Mu insertions per 100 bp as a function of distance outside Mu during EST (top row) and LST (bottom row). The distances reported are combined for both the left and right ends of Mu (see Fig. S4 for individual ends). For bulk DNA, the average number of insertions into a 100 bp region is nearly 25 insertions per 5 million reads during EST, and is indicated by the solid blue line. The shaded blue area is the standard deviation for the number of insertions expected within 100 bp. For OPL-Mu (bottom row), with only one location reporting, the bulk DNA average is around 6 insertions per 100 nucleotides

During EST, WT Mu does not transpose within 1.5 kb outside each of the starting Mu positions, consistent with the *cis*-immunity phenomenon (Fig. 4a, bottom row of all plots). That the absence of transposition in this region is not due to an intrinsic resistance to insertion within this DNA, is seen from the pooled profiles of the other prophages for the same region (WT pool). Figure 4b examines this pattern in greater detail. For EST (Fig. 4b, top left), three distinct insertion phases can be observed: 1) a low probability initial phase, where there is a slow increase in the number of insertions starting around 2 kb outside both Mu ends, 2) a boundary phase exhibiting a sharp increase in insertions around 5 kb, and 3) a bulk phase, reaching the average number of insertions for bulk DNA beginning around 7 kb. This pattern was symmetrical for individual ends (Fig. S4). For LST (OPL-Mu; Fig. 4b, bottom left), three similar phases were observed, although the initial phase extended past 5 kb.

ΔMuB insertion patterns for starting prophages and the pooled profiles of other prophages (ΔMuB pool) are shown in Fig. 4a. The insertion profiles outside Mu ends were not only different from WT, but also different between EST and LST. During EST (Fig. 4b, top right and Fig. S4), only two insertion phases were observed: 1) an extended linear phase starting between 500 to 600 bp, and 2) a bulk phase, reaching the average number of insertions for bulk DNA at around 7 kb, similar to WT. During LST, *cis*-immunity was completely abrogated in both ΔMuB OPL-Mu alone and in all six ΔMuB prophages combined (Fig. 4b bottom middle and right), in contrast to WT OPL-Mu where the immunity stayed intact (Fig. 4b, bottom left). For ΔMuB LST, there was only a short linear phase where insertions started at 98 bp, reaching bulk efficiency early, starting at around 2 kb. We attribute the difference in the EST and LST ΔMuB insertion patterns to the lower transposition efficiency of ΔMuB prophages, which did not provide sufficient opportunity to sample nearby space during EST, but allowed saturation of the *cis* region from distant Mu’s generated by increased Mu copy numbers during LST. We conclude that MuB is indeed responsible for *cis*-immunity in vivo.

The previously described ratchet-model suggests that intrinsically clustered MuA would hydrolyze proximal MuB-ATP during dynamic loop formation due to Brownian motion [25]. As proposed by this model, the distinct tri-phase WT pattern would come from the rapid dissociation of proximal MuB, leading to distal sites (5 kb away) being captured more efficiently for integration. We propose that the two-phase pattern of target selection in EST ΔMuB is actually the measurement of dynamic loop formation in vivo, the loops being ~ 7 kb in size. Naively assuming that MuB binding doesn’t alter the rates of loop formation, the stable 7 kb loop formation would remain consistent between WT and ΔMuB.

What is the importance of *cis*-immunity in the life of Mu? Avoiding insertion into regions flanking Mu ends would avoid destroying flanking Mu copies when packaging begins, since the DNA packaging machinery resects on average 100 bp of host DNA flanking the left end and 1.5 kb of DNA flanking the right end. Even though Mu samples the *E. coli* genome extensively in a distance-independent manner (Fig. 2) [29], loss of even a small fraction of *cis* Mus during packaging might impact fitness. It is possible that *cis*-immunity is an evolutionary remnant of MuB- and MuA-like functions in an ancestral transposon, where additional partner proteins directed transposition to specific sites. For example, Tn3 and Tn7 exhibit target immunity much further than Mu [22, 52, 53]. Tn7 has two proteins TnsB and TnsC that are thought to play roles similar to MuB and MuA respectively. Tn7 has two partner proteins, TnsD and TnsE, that promote different target choices. Han and Mizuuchi [25] discuss how the Mu *cis*-immunity system may have evolved from a Tn7-type target site search. Mu apparently discarded these partners during an evolutionary trajectory more suited to its viral lifestyle, acquiring features that unfettered its ability to choose.

### MuB is only partially responsible for Mu genome immunity

**Fig. 5:**
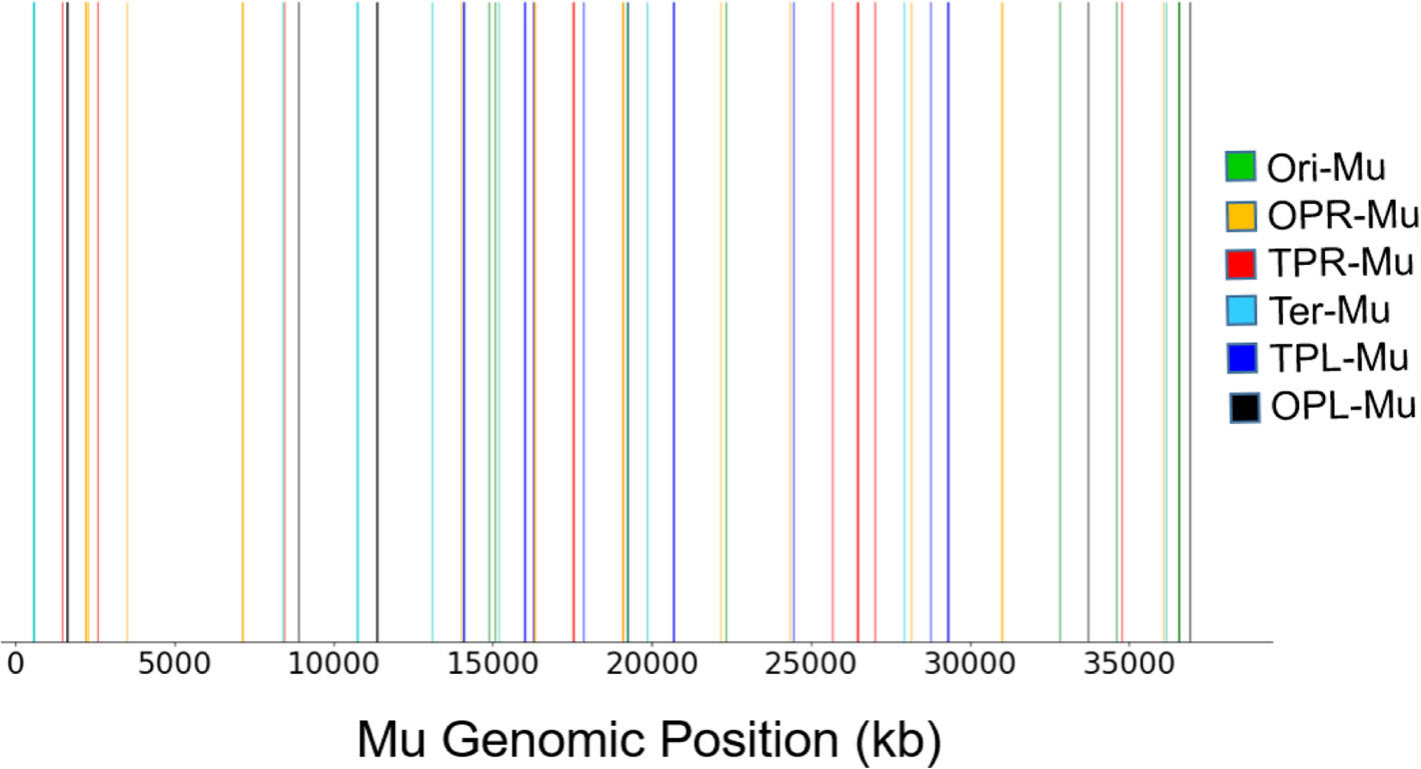
ΔMuB prophages exhibit very low levels of self-integration. WT and ΔMuB prophage transpositions during LST were analyzed for novel Mu junctions that would indicate Mu self-integration (SI). Out of ~ 10 million insertions, no instances of SI were observed in WT (data not shown), and 85 SI sites were observed in ΔMuB prophages. These sites are plotted along the Mu genomic position. Each insertion is color-coded to correspond to the prophage that specific insertion belongs to

The *cis*-immunity phenomenon depends on MuB removal from DNA adjacent to and outside Mu ends. By contrast, inside Mu, MuB was observed to bind strongly during the lytic cycle, implicating a role for bound MuB in Mu genome immunity [27]. In the EST insertion data shown in Fig. 4a, there were no observable self-insertions (SI) in either WT or ΔMuB (the latter have 1.5x the depth of sequence reads compared to WT). SI was also not detected in the EST data for 35 WT prophages reported earlier [29]. To determine if this immunity is still intact at the end of the lytic cycle, we examined LST counts in the two prophage populations (Fig. 5). The WT OPL-Mu was still immune to SI (not shown), but the ΔMuB prophages, which have higher copy numbers in LST, now showed evidence of self-insertion. However, out of 90 Million Mu targeted reads from deep sequencing, 85 instances of SI were observed, spread across all 6 starting ΔMuB prophages. We conclude that, unlike *cis*-immunity which is completely abrogated in the absence of MuB (Fig. 4b bottom row), genome immunity is only faintly violated. Therefore, the bulk of genome immunity is determined by factors other than MuB.

Mu ends (L and R) define a boundary separating two modes of MuB binding and immunity [27]. We had proposed that Mu genome immunity arises from a special structure that Mu adopts, aided by both specific Mu sequences and by general cellular nucleoid associated proteins (NAPs). In the center of the genome is the strong gyrase-binding site (SGS), which is essential for Mu replication in vivo and is believed to function by influencing efficient synapsis of the Mu ends [54–56]. The SGS is thought to act by localizing the 37 kb Mu prophage DNA into a single loop of plectonemically supercoiled DNA upon binding of DNA gyrase to the site. We had proposed that an SGS-generated Mu loop, sealed off at the Mu ends by either the transpososome or NAPs, serves as a scaffold for nucleating MuB filaments in the Mu interior, providing a barrier to Mu integration. Evidence for a separate, stable prophage Mu domain, bounded by the proximal location of Mu L and R ends, was indeed obtained [28]. Formation/maintenance of the Mu domain was dependent on SGS, the Mu L end, MuB protein, and the *E. coli* NAPs IHF, Fis and HU. Of these components, SGS is essential for Mu transposition in vivo [57, 58], hence its contribution to Mu genome immunity cannot be assessed. To examine the contribution of the NAPs, we analyzed our published data where we had monitored Mu transposition in all NAP mutants of *E. coli* (these were collected during EST) [29]. We observed no instances of Mu self-transposition in any of the NAP deletions examined.

### Summary

MuB is critical for Mu’s ability to efficiently capture targets for transposition. We show in this study that besides enabling efficient targeting, MuB also makes refractory targets more facile, likely by displacing bound proteins. By weakening/altering boundary features that demarcates the Ter region, MuB allows Mu to access Ter more readily. Transposition patterns in the absence of MuB have allowed us to more accurately measure the Ter boundaries, revealing that this region is larger than previously estimated. Perhaps in a similar manner, MuB also provides access to targets engaged in transcription/translation. We have mapped the range of *cis*-immunity more accurately, and show that it persists well into the lytic cycle for WT prophages, but is abolished in ΔMuB strains. We show that Mu genome immunity also persists through the lytic cycle for WT prophages, and is only rarely infringed upon in ΔMuB prophages, showing conclusively the distinction between these two forms of immunity. There is clearly more to be learned about what enables genome immunity.

## Materials and methods

### Strain information and growth conditions

All experimental strains are derivatives of MG1655 and listed in the strains table. Prophage gene deletions were introduced into specific prophages using P1 transduction and kanamycin resistance selection. Cells were propagated by shaking at 30 °C in M9-Cas minimal media (0.2% casamino acids, 0.2% glucose, 100 μg/mL thiamine) and appropriate antibiotics for selection.

### Transposition

Prophage transposition was induced by temperature shifting to 42 °C for the appropriate time before harvesting genomic DNA. Early stage transposition (EST) experiments were accomplished by a 15 min temperature shift to capture one transposition event in WT cells as determined in a previous study [29]. Late stage transposition (LST) experiments were done by a temperature shift for 2 h. At the end of this time, cell lysis had occurred for WT prophages but not for ΔMuB prophages. Lysogen genomic DNA was purified using a commercially available gDNA purification kit (Wizard, Promega). gDNA samples were stored at − 20 °C in a 10 mM Tris pH 8.0, 1 mM EDTA buffer until ready for target enrichment.

### Target enrichment

Oligonucleotide primer sequences are provided in Table S1. Primer y-link1 has a hand mixed random 6 nucleotide barcode to identify PCR duplicates in sequencing. Y-link adapters were annealed by mixing equivalent amounts of primers y-link1 and y-link2 at room temperature and heating to 95 °C then cooled down to 4 °C using a temperature ramp of 1 °C per second. Genomic DNA was digested with the frequent cutter HinPI (NEB) and then ligated with the y-link adapter using a Quick Ligation Kit (NEB). The ligation product was purified using magnetic beads (Axygen). Mu insertion targets were enriched, by PCR amplification of the ligation product using y-link_primer and Mu_L31, an initial melting temp of 95 °C for 1 min and 8 cycles of 95 °C for 20 s, 68 °C for 20 s, 72 °C for 1 min. A final extension of 72 °C was added for 5 min. The PCR product was purified using magnetic beads (Axygen) and frozen at − 20 °C until ready for sequencing.

### Genomic sequencing

Target enriched samples were submitted to the Genomic Sequencing and Analysis Facility (GSAF) at UT Austin for sequencing. Libraries were prepped by GSAF using the facility’s low-cost high throughput method. Sequencing was done on an Illumina NextSeq 500 platform using 2X150 paired ends targeting 10 to 15 million reads. All sequencing data discussed in this work is available at https://www.ncbi.nlm.nih.gov/sra/PRJNA597349.

### Identifying Mu insertion locations

Mu transposition targets were identified using lab software entitled Mu Analysis of Positions from Sequencing (MAPS) as described earlier [29]. MAPS has been modified since initial publication to provide nucleotide precision for target enriched samples and provide self-insertion information. In short, MAPS now identifies Mu-host junctions by identifying the 12-mer sequence unique to the y-link adapter used in target enrichment. The current version of MAPS is available for download at https://github.com/dmwalker/MuSeq.

## Supplementary information

**Additional file 1: Figure S1.** Mu transposition outlines several features of the rRNA operons. EST prophages were pooled to analyze the frequency of Mu insertions into the entire rrn operon, for all 7 operons, for both WT (bottom rows) and ΔMuB (top rows) prophages. Insertion maps start at the TSS of the operon and continue for 5.3 kb. Operon maps are provided as a schematic on top, showing the leading 16 s RNA-encoding segment, followed by coding sequence (CDS) of an intervening tRNA, and finally the 23 s RNA-encoding segment. Each CDS in the operon is marked by a blue line that terminates in a flat head. The rrnD and rrnG operons are located on the (−) strand of DNA, while the remaining 5 are on the (+) strand. ΔMuB patterns generally follow similar trends to the WT prophage, but with reduced efficacy to insert anywhere within the rRNA operon. **Figure S2.** Mu does not transpose easily into tRNA coding regions. The x-axis provides the relative genomic position with respect to the tRNA labeled on the y-axis, and covers a 400 bp span. The + 1 position indicates the first nucleotide in the matured tRNA sequence. The preprocessed 5′ leader coding region would be encompassed in the region between − 200 to + 1. The e position is + 75 bp from the TSS and is the typical size of mature tRNA. The + 150 region is 150 bp from the TSS. For each of the 86 tRNA genes, the number of Mu insertions in and around the gene are tabulated for both the WT and ΔMuB prophages during EST. The transcriptional propensity is nucleotide level resolution of the degree of transcription for that particular nucleotide [48]. A higher number means higher degree of transcription. Two genes of interest (red asterisks) are enlarged on the bottom. There are no Mu transpositions in regions of high transcription within these genes. **Figure S3.** Frequency of consensus target sequences for WT and ΔMuB prophage insertions across the E. coli genome. A. The genome for MG1655 from genbank (genid: 545778205) was partitioned into 200 equally sized bins, and the number of times the 5′-CYSRG-3‘ sequence and it’s reverse compliment appeared on the + strand in each bin was tabulated. B. The number of Mu insertions for each consensus sequence was calculated for both the WT (left) and ΔMuB prophages (right). The number of insertions reported is for the consensus sequence as written and the corresponding reverse complement. There are 1024 possible pentamers for Mu to insert, and the sequence identifier ‘other’ accounts for the 1016 sequences not covered by the ‘CYSRG’ consensus sequences and their reverse compliment. **Figure S4.** Insertion patterns outside Mu ends are nearly symmetrical for the left and right ends of Mu. The frequency of Mu insertions per 100 bp as a function of distance from Mu is plotted individually for each end, the combined data shown in Fig. 4. For WT Mu, the first Mu insertion on the left side occurred at 1.6 kb from the left end, while ΔMuB insertions started at 529 bp. The right end insertions of WT Mu started around 3.1 kb and at 544 bp for ΔMuB prophages. WT cis-immunity shows a sharp decline around 5 kb for both the right and left ends of Mu, while the ΔMuB prophages show a steady increase in insertions away from the initial prophage.

**Additional file 2: Table S1.** Oligonucleotides used in this study.

## Data Availability

All strains generated in this study are available without restriction. The sequencing data presented in this paper can be accessed on the SRA database under the project number PRJNA597349. Software used to analyze the sequencing data can be accessed by github (DOI 10.5281/zenodo.3762807).

## References

[1] Symonds N, Toussaint A, Van de Putte P, Howe MM (1987). Phage Mu.

[2] Craig NL (2015). Mobile DNA III.

[3] Harshey RM, Craig NL (2015). Transposable phage Mu. In Mobile DNA III.

[4] Mizuuchi K (1992). Transpositional recombination: mechanistic insights from studies of mu and other elements. Annu Rev Biochem.

[5] Nakai H, Doseeva V, Jones JM (2001). Handoff from recombinase to replisome: insights from transposition. Proc Natl Acad Sci U S A.

[6] Bukhari AI, Taylor AL (1975). Influence of insertions on packaging of host sequences covalently linked to bacteriophage mu DNA. Proc Natl Acad Sci U S A.

[7] Kahmann R, Kamp D (1979). Nucleotide sequences of the attachment sites of bacteriophage mu DNA. Nature.

[8] Manna D, Deng S, Breier AM, Higgins NP (2005). Bacteriophage mu targets the trinucleotide sequence CGG. J Bacteriol.

[9] Chaconas G, Harshey RM, Craig NL, Craigie R, Gellert M, Lambowitz AM (2002). Transposition of phage Mu DNA. In Mobile DNA II.

[10] Greene EC, Mizuuchi K (2004). Visualizing the assembly and disassembly mechanisms of the MuB transposition targeting complex. J Biol Chem.

[11] Mizuno N, Dramicanin M, Mizuuchi M, Adam J, Wang Y, Han YW, Yang W, Steven AC, Mizuuchi K, Ramon-Maiques S (2013). MuB is an AAA+ ATPase that forms helical filaments to control target selection for DNA transposition. Proc Natl Acad Sci U S A.

[12] Dramicanin M, Lopez-Mendez B, Boskovic J, Campos-Olivas R, Ramon-Maiques S (2015). The N-terminal domain of MuB protein has striking structural similarity to DNA-binding domains and mediates MuB filament-filament interactions. J Struct Biol.

[13] Montano SP, Pigli YZ, Rice PA (2012). The mu transpososome structure sheds light on DDE recombinase evolution. Nature.

[14] Fuller JR, Rice PA (2017). Target DNA bending by the mu transpososome promotes careful transposition and prevents its reversal. Elife.

[15] Craig NL (1997). Target site selection in transposition. Annu Rev Biochem.

[16] Mizuuchi M, Mizuuchi K (1993). Target site selection in transposition of phage mu. Cold Spring Harb Symp Quant Biol.

[17] Haapa-Paananen S, Rita H, Savilahti H (2002). DNA transposition of bacteriophage Mu. A quantitative analysis of target site selection in vitro. J Biol Chem.

[18] Stellwagen AE, Craig NL (1997). Avoiding self: two Tn7-encoded proteins mediate target immunity in Tn7 transposition. EMBO J.

[19] Skelding Z, Queen-Baker J, Craig NL (2003). Alternative interactions between the Tn7 transposase and the Tn7 target DNA binding protein regulate target immunity and transposition. EMBO J.

[20] Nicolas E, Lambin M, Hallet B (2010). Target immunity of the Tn3-family transposon Tn4430 requires specific interactions between the transposase and the terminal inverted repeats of the transposon. J Bacteriol.

[21] Lambin M, Nicolas E, Oger CA, Nguyen N, Prozzi D, Hallet B (2012). Separate structural and functional domains of Tn4430 transposase contribute to target immunity. Mol Microbiol.

[22] Lee CH, Bhagwat A, Heffron F (1983). Identification of a transposon Tn3 sequence required for transposition immunity. Proc Natl Acad Sci U S A.

[23] Adzuma K, Mizuuchi K (1988). Target immunity of mu transposition reflects a differential distribution of mu B protein. Cell.

[24] Greene EC, Mizuuchi K (2002). Target immunity during Mu DNA transposition. Transpososome assembly and DNA looping enhance MuA-mediated disassembly of the MuB target complex. Mol Cell.

[25] Han YW, Mizuuchi K (2010). Phage mu transposition immunity: protein pattern formation along DNA by a diffusion-ratchet mechanism. Mol Cell.

[26] Manna D, Higgins NP (1999). Phage mu transposition immunity reflects supercoil domain structure of the chromosome. Mol Microbiol.

[27] Ge J, Lou Z, Harshey RM (2010). Immunity of replicating mu to self-integration: a novel mechanism employing MuB protein. Mob DNA.

[28] Saha RP, Lou Z, Meng L, Harshey RM (2013). Transposable prophage Mu is organized as a stable chromosomal domain of E coli. PLoS Genet.

[29] Walker DM, Freddolino PL, Harshey RM (2020). A well-mixed E coli genome: widespread contacts revealed by tracking Mu transposition. Cell.

[30] Harshey RM, Symonds N, Toussaint A, Van de Putte P, Howe MM (1987). Integration of infecting Mu DNA. In Phage Mu.

[31] Valens M, Penaud S, Rossignol M, Cornet F, Boccard F (2004). Macrodomain organization of the *Escherichia coli* chromosome. EMBO J.

[32] Mercier R, Petit MA, Schbath S, Robin S, El Karoui M, Boccard F, Espeli O (2008). The MatP/matS site-specific system organizes the terminus region of the E. coli chromosome into a macrodomain. Cell.

[33] Nolivos S, Upton AL, Badrinarayanan A, Muller J, Zawadzka K, Wiktor J, Gill A, Arciszewska L, Nicolas E, Sherratt D (2016). MatP regulates the coordinated action of topoisomerase IV and MukBEF in chromosome segregation. Nat Commun.

[34] Makela J, Sherratt DJ (2020). Organization of the Escherichia coli chromosome by a MukBEF axial Core. Mol Cell.

[35] Cagliero C, Grand RS, Jones MB, Jin DJ, O'Sullivan JM (2013). Genome conformation capture reveals that the Escherichia coli chromosome is organized by replication and transcription. Nucleic Acids Res.

[36] Lioy VS, Cournac A, Marbouty M, Duigou S, Mozziconacci J, Espeli O, Boccard F, Koszul R (2018). Multiscale structuring of the E coli chromosome by nucleoid-associated and condensin proteins. Cell.

[37] Manna D, Breier AM, Higgins NP (2004). Microarray analysis of transposition targets in *Escherichia coli*: the impact of transcription. Proc Natl Acad Sci U S A.

[38] Ge J, Lou Z, Cui H, Shang L, Harshey RM (2011). Analysis of phage mu DNA transposition by whole-genome *Escherichia coli* tiling arrays reveals a complex relationship to distribution of target selection protein B, transcription and chromosome architectural elements. J Biosci.

[39] Mouslim C, Hughes KT (2014). The effect of cell growth phase on the regulatory cross-talk between flagellar and Spi1 virulence gene expression. PLoS Pathog.

[40] Nonaka G, Blankschien M, Herman C, Gross CA, Rhodius VA (2006). Regulon and promoter analysis of the E. coli heat-shock factor, sigma32, reveals a multifaceted cellular response to heat stress. Genes Dev.

[41] Mendoza-Vargas A, Olvera L, Olvera M, Grande R, Vega-Alvarado L, Taboada B, Jimenez-Jacinto V, Salgado H, Juarez K, Contreras-Moreira B (2009). Genome-wide identification of transcription start sites, promoters and transcription factor binding sites in *E. coli*. PLoS One.

[42] Muller-Hill B (1996). The lac operon.

[43] Keseler IM, Mackie A, Santos-Zavaleta A, Billington R, Bonavides-Martinez C, Caspi R, Fulcher C, Gama-Castro S, Kothari A, Krummenacker M (2017). The EcoCyc database: reflecting new knowledge about Escherichia coli K-12. Nucleic Acids Res.

[44] Condon C, Philips J, Fu ZY, Squires C, Squires CL (1992). Comparison of the expression of the seven ribosomal RNA operons in Escherichia coli. EMBO J.

[45] Bartlett MS, Gaal T, Ross W, Gourse RL (2000). Regulation of rRNA transcription is remarkably robust: FIS compensates for altered nucleoside triphosphate sensing by mutant RNA polymerases at Escherichia coli rrn P1 promoters. J Bacteriol.

[46] Maeda M, Shimada T, Ishihama A (2015). Strength and regulation of seven rRNA promoters in Escherichia coli. PLoS One.

[47] McDonald MJ, Chou CH, Swamy KB, Huang HD, Leu JY (2015). The evolutionary dynamics of tRNA-gene copy number and codon-use in E coli. BMC Evol Biol.

[48] Scholz SA, Diao R, Wolfe MB, Fivenson EM, Lin XN, Freddolino PL (2019). High-resolution mapping of the Escherichia coli chromosome reveals positions of high and low transcription. Cell Syst.

[49] Tan X, Mizuuchi M, Mizuuchi K (2007). DNA transposition target immunity and the determinants of the MuB distribution patterns on DNA. Proc Natl Acad Sci U S A.

[50] Ge J, Harshey RM (2008). Congruence of in vivo and in vitro insertion patterns in hot *E. coli* gene targets of transposable element mu: opposing roles of MuB in target capture and integration. J Mol Biol.

[51] Yanagihara K, Mizuuchi K (2002). Mismatch-targeted transposition of mu: a new strategy to map genetic polymorphism. Proc Natl Acad Sci U S A.

[52] Craig N (2002). Tn7.

[53] Peters JE, Craig NL (2015). Tn7. In Mobile DNA III.

[54] Pato ML (1994). Central location of the mu strong gyrase binding site is obligatory for optimal rates of replicative transposition. Proc Natl Acad Sci U S A.

[55] Pato ML, Banerjee M (1996). The mu strong gyrase-binding site promotes efficient synapsis of the prophage termini. Mol Microbiol.

[56] Pato ML, Howe MM, Higgins NP (1990). A DNA gyrase-binding site at the center of the bacteriophage mu genome is required for efficient replicative transposition. Proc Natl Acad Sci U S A.

[57] Pato ML, Karlok M, Wall C, Higgins NP (1995). Characterization of mu prophage lacking the central strong gyrase binding site: localization of the block in replication. J Bacteriol.

[58] Pato ML (2004). Replication of mu prophages lacking the central strong gyrase site. Res Microbiol.

