# Supplementary material for: Deep sequencing reveals new roles for MuB in transposition immunity and target-capture, and redefines the insular Ter region of E. coli
Source: Mob DNA. 2020 Jul 9;11:26. doi: 10.1186/s13100-020-00217-9 (PMC7350765; doi:10.1186/s13100-020-00217-9)
Supplement: Supplementary file 1 — Additional file 1: Figure S1. Mu transposition outlines several features of the rRNA operons. EST prophages were pooled to analyze the frequency of Mu insertions into the entire rrn operon, for all 7 operons, for both WT (bottom rows) and ΔMuB (top rows) prophages. Insertion maps start at the TSS of the operon and continue for 5.3 kb. Operon maps are provided as a schematic on top, showing the leading 16 s RNA-encoding segment, followed by coding sequence (CDS) of an intervening tRNA, and finally the 23 s RNA-encoding segment. Each CDS in the operon is marked by a blue line that terminates in a flat head. The rrnD and rrnG operons are located on the (−) strand of DNA, while the remaining 5 are on the (+) strand. ΔMuB patterns generally follow similar trends to the WT prophage, but with reduced efficacy to insert anywhere within the rRNA operon. Figure S2. Mu does not transpose easily into tRNA coding regions. The x-axis provides the relative genomic position with respect to the tRNA labeled on the y-axis, and covers a 400 bp span. The + 1 position indicates the first nucleotide in the matured tRNA sequence. The preprocessed 5′ leader coding region would be encompassed in the region between − 200 to + 1. The e position is + 75 bp from the TSS and is the typical size of mature tRNA. The + 150 region is 150 bp from the TSS. For each of the 86 tRNA genes, the number of Mu insertions in and around the gene are tabulated for both the WT and ΔMuB prophages during EST. The transcriptional propensity is nucleotide level resolution of the degree of transcription for that particular nucleotide [48]. A higher number means higher degree of transcription. Two genes of interest (red asterisks) are enlarged on the bottom. There are no Mu transpositions in regions of high transcription within these genes. Figure S3. Frequency of consensus target sequences for WT and ΔMuB prophage insertions across the E. coli genome. A. The genome for MG1655 from genbank (genid: 545778205) was parti [file 13100_2020_217_MOESM1_ESM.docx]

**Supplementary Figures**

**
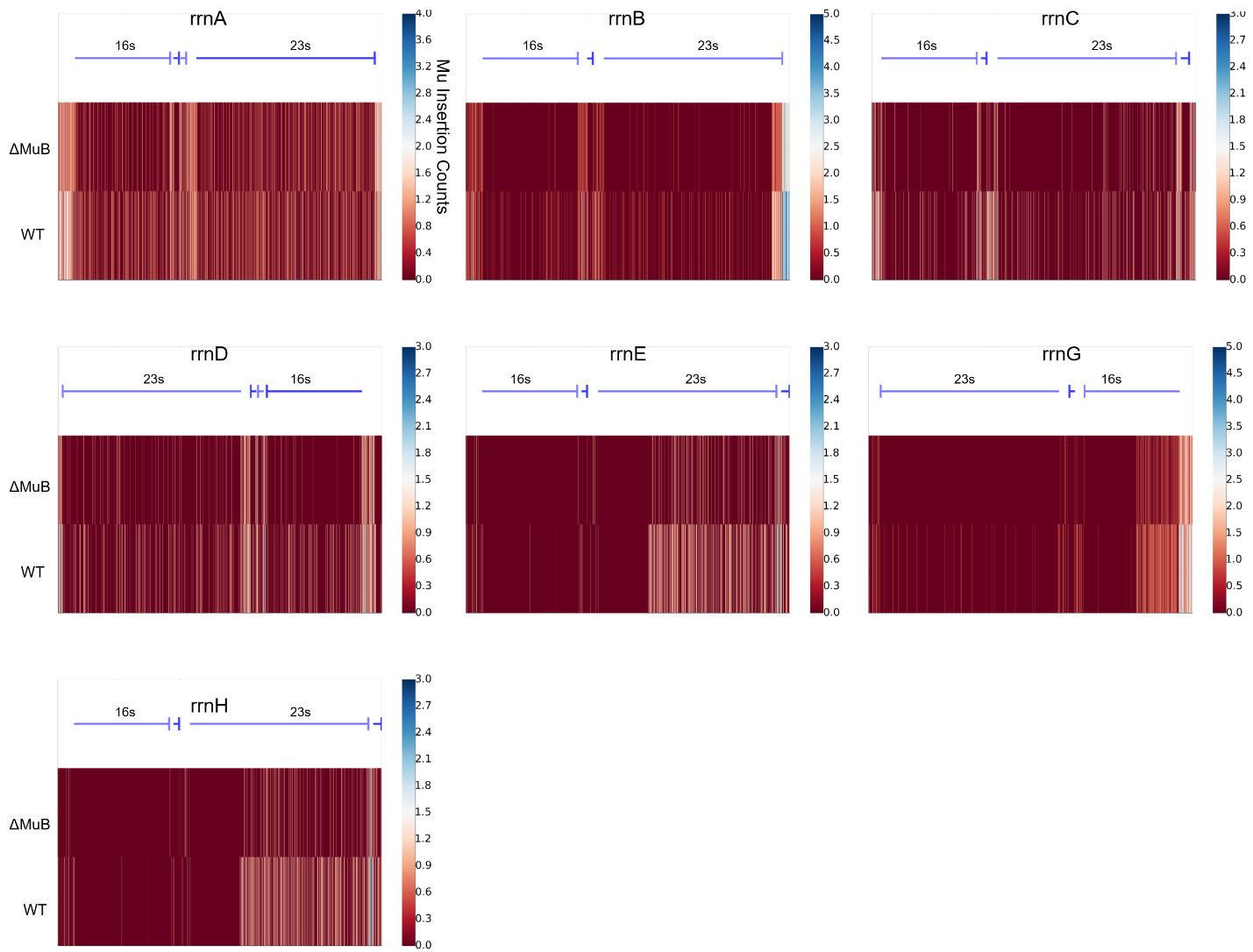
**

**Figure S1. Mu transposition outlines several features of the rRNA operons.** EST prophages were pooled to analyze the frequency of Mu insertions into the entire *rrn* operon, for all 7 operons, for both WT (bottom rows) and ΔMuB (top rows) prophages. Insertion maps start at the TSS of the operon and continue for 5.3 kb. Operon maps are provided as a schematic on top, showing the leading 16s RNA-encoding segment, followed by coding sequence (CDS) of an intervening tRNA, and finally the 23s RNA-encoding segment. Each CDS in the operon is marked by a blue line that terminates in a flat head. The *rrnD* and *rrnG* operons are located on the (-) strand of DNA, while the remaining 5 are on the (+) strand. ΔMuB patterns generally follow similar trends to the WT prophage, but with reduced efficacy to insert anywhere within the rRNA operon.


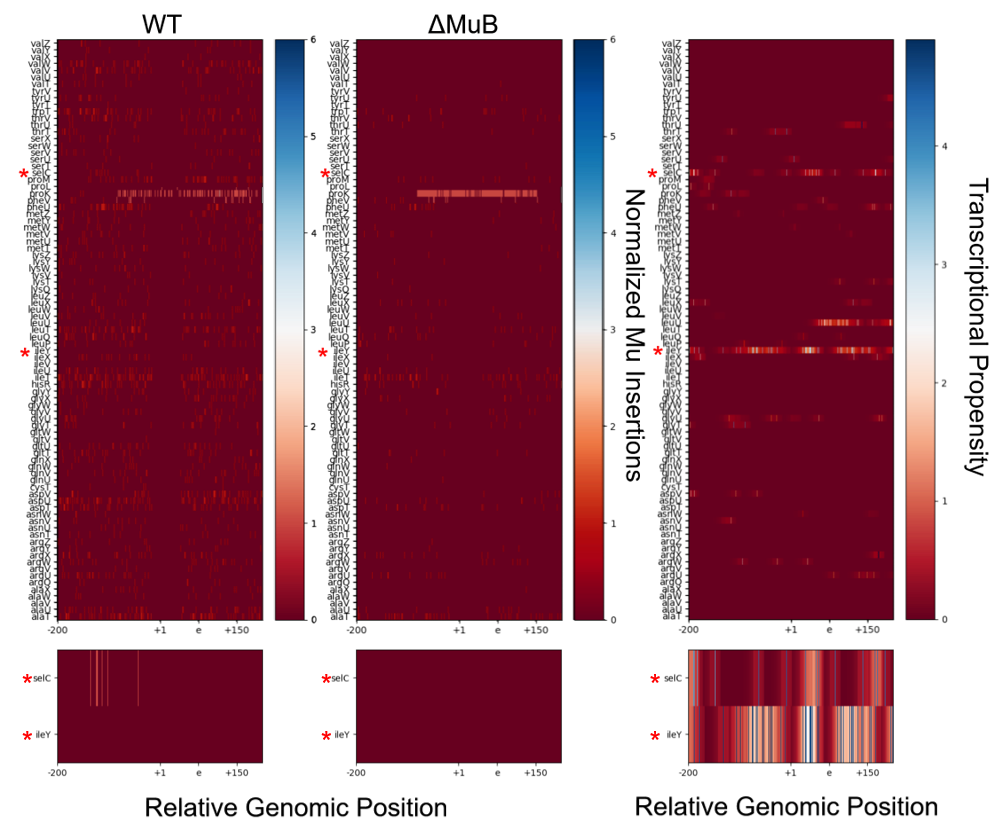


**Figure S2. Mu does not transpose easily into tRNA coding regions.** The x-axis provides the relative genomic position with respect to the tRNA labeled on the y-axis, and covers a 400 bp span. The +1 position indicates the first nucleotide in the matured tRNA sequence. The preprocessed 5' leader coding region would be encompassed in the region between -200 to +1. The e position is +75 bp from the TSS and is the typical size of mature tRNA. The +150 region is 150 bp from the TSS. For each of the 86 tRNA genes, the number of Mu insertions in and around the gene are tabulated for both the WT and ΔMuB prophages during EST. The transcriptional propensity is nucleotide level resolution of the degree of transcription for that particular nucleotide [48]. A higher number means higher degree of transcription. Two genes of interest (red asterisks) are enlarged on the bottom. There are no Mu transpositions in regions of high transcription within these genes.

**
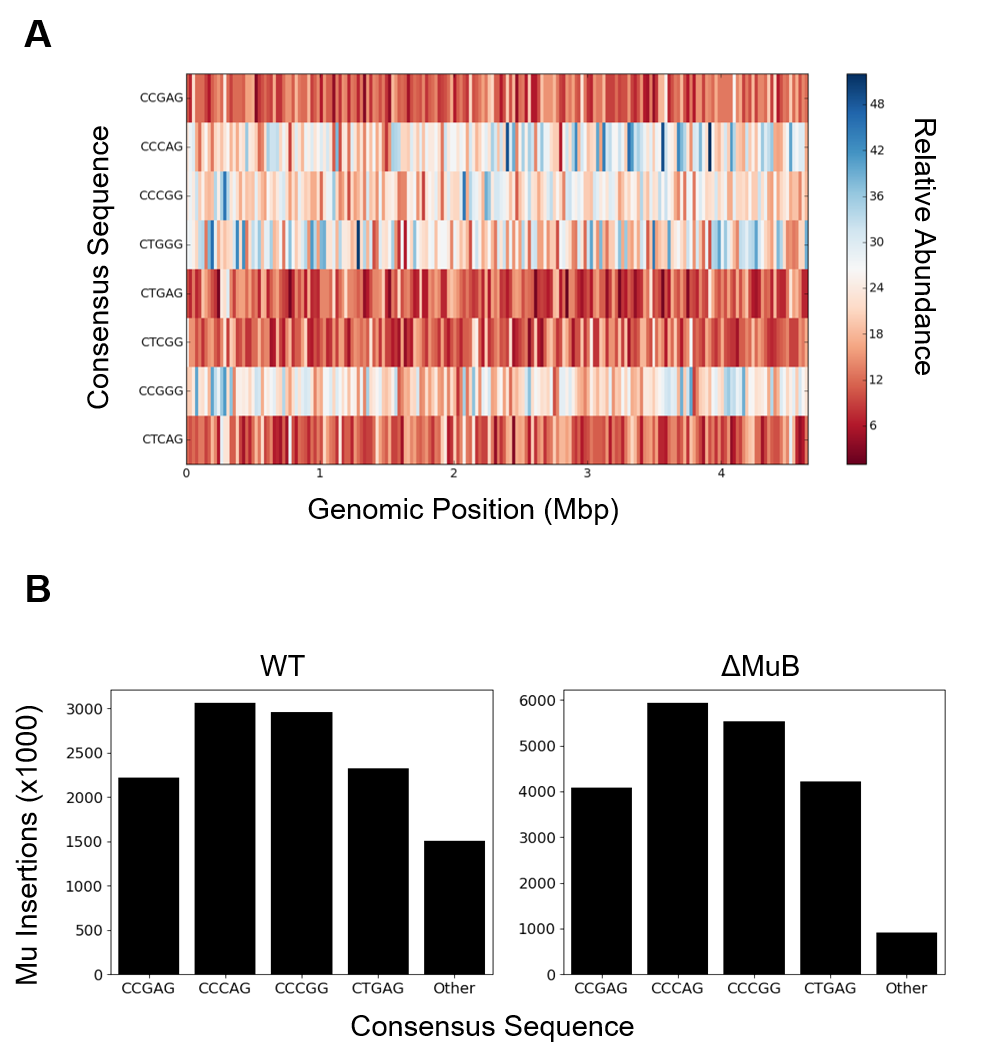
**

**Figure S3.** **Frequency of consensus target sequences for WT and ΔMuB prophage insertions across the *E. coli* genome.** **A.** The genome for MG1655 from genbank (genid: 545778205) was partitioned into 200 equally sized bins, and the number of times the 5’-CYSRG-3‘ sequence and it’s reverse compliment appeared on the + strand in each bin was tabulated. **B.** The number of Mu insertions for each consensus sequence was calculated for both the WT (left) and ΔMuB prophages (right). The number of insertions reported is for the consensus sequence as written and the corresponding reverse complement. There are 1024 possible pentamers for Mu to insert, and the sequence identifier ‘other’ accounts for the 1016 sequences not covered by the ‘CYSRG’ consensus sequences and their reverse compliment.


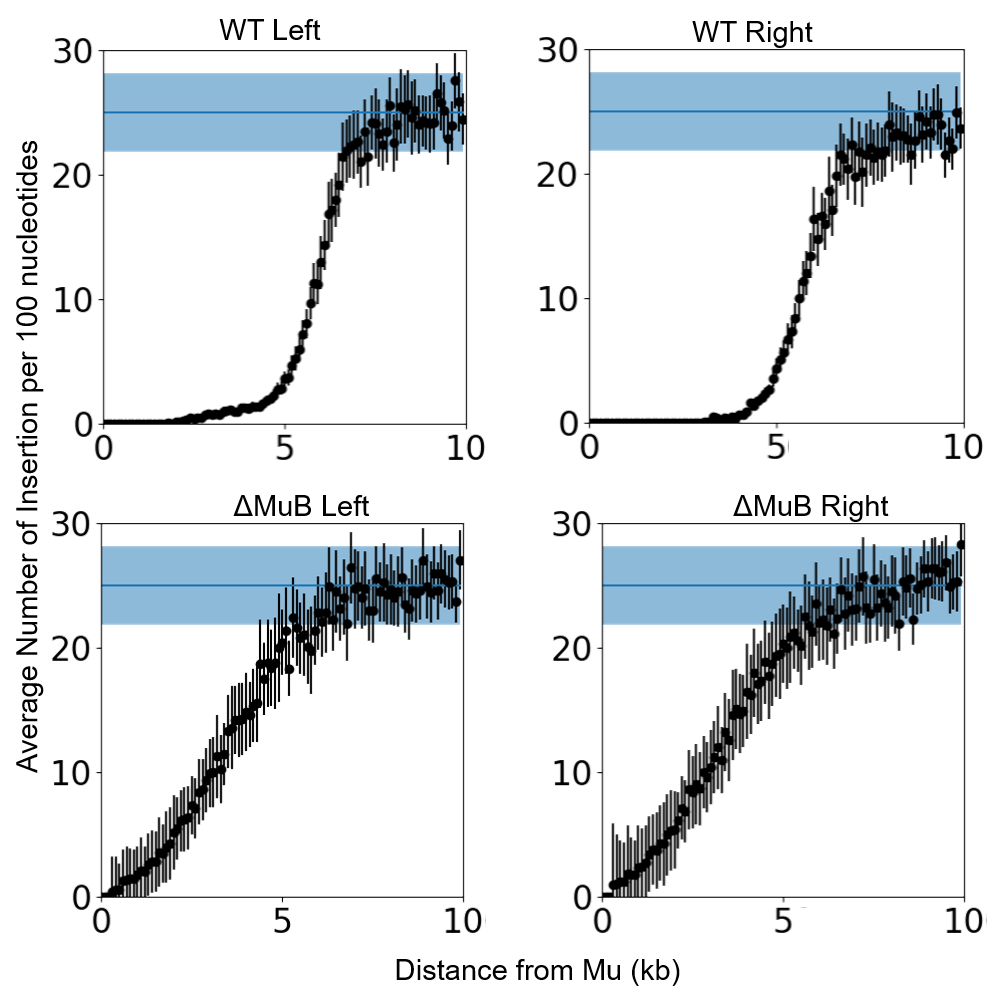


**Figure S4**. **Insertion patterns outside Mu ends are nearly symmetrical for the left and right ends of Mu.** The frequency of Mu insertions per 100 bp as a function of distance from Mu is plotted individually for each end, the combined data shown in Fig. 4. For WT Mu, the first Mu insertion on the left side occurred at 1.6 kb from the left end, while ΔMuB insertions started at 529 bp. The right end insertions of WT Mu started around 3.1 kb and at 544 bp for ΔMuB prophages. WT *cis*-immunity shows a sharp decline around 5 kb for both the right and left ends of Mu, while the ΔMuB prophages show a steady increase in insertions away from the initial prophage.
