# Supplementary material for: Deep sequencing reveals new roles for MuB in transposition immunity and target-capture, and redefines the insular Ter region of E. coli
Source: Mob DNA. 2020 Jul 9;11:26. doi: 10.1186/s13100-020-00217-9 (PMC7350765; doi:10.1186/s13100-020-00217-9)
Supplement: Supplementary file 2 — Additional file 2: Table S1. Oligonucleotides used in this study. [file 13100_2020_217_MOESM2_ESM.docx]

**Table S1. Oligonucleotides used in this study**

| REAGENT or RESOURCE | SOURCE | IDENTIFIER |
| --- | --- | --- |
| Oligonucleotides | | |
| TATACTGCTCGAATTCAGAGCTTCNNNNNNAAGCTTCTAACGATGTACGGGGACAG | This Study | y-link1 |
| CGCTGTCCCCGTACATCGTTAGAAGCTTGCTAACCTGAT | This Study | y-link2 |
| CTGCTCGAATTCAGAGCTTC | This Study | y-link_primer |
| CCGGGAGGACATTGGATTATTCGGGATCTG | This Study | Mu_L31 |
